# Prognostic value of prognostic nutritional index in patients with ovarian cancer: a systematic review and meta-analysis

**DOI:** 10.3389/fonc.2025.1693559

**Published:** 2026-01-06

**Authors:** Tao Zang, Bintao Huang, Qinggao Wang

**Affiliations:** 1 The First Clinical Medical College, Guangxi University of Chinese Medicine, Nanning, Guangxi Zhuang Autonomous Region, China; 2 Department of Cardiology, First Affiliated Hospital, Guangxi University of Chinese Medicine, Nanning, Guangxi Zhuang Autonomous Region, China

**Keywords:** meta-analysis, ovarian cancer, overall survival, prognostic value of survival, progression-free survival, the prognostic nutritional index

## Abstract

**Background:**

Accumulating evidence indicates an association between the prognostic nutritional index (PNI) and prognosis in patients with ovarian cancer (OC). However, the conclusions drawn from current studies remain controversial.

**Methods:**

We performed a systematic literature search in PubMed, Embase, Web of Science, and the Cochrane Library, covering the period from each database’s inception up to July 8, 2025, to identify studies investigating the relationship between PNI and clinical outcomes. Eligible studies were identified and selected based on predefined inclusion and exclusion criteria. Primary outcomes included progression-free survival (PFS), overall survival (OS), cancer-specific survival (CSS), and disease-specific survival (DSS), which were quantified using hazard ratios (HR) with their respective 95% confidence intervals (CI).

**Results:**

We analyzed thirteen cohort studies involving 5,129 patients. Meta-analysis findings demonstrated an association between reduced PNI and poorer PFS (HR = 1.59, 95% CI: 1.24–2.03; p=0.0002) and curtailed OS (HR = 1.72, 95% CI: 1.36–2.18; p<0.00001), whereas no such correlation was observed in the DSS (HR = 1.88, 95% CI: 0.70–5.10; p=0.21) and CSS (HR = 1.91, 95% CI: 0.81–4.52; p=0.14) analyses.

**Conclusion:**

A low PNI is associated with shortened OS and PFS in OC patients. PNI thus serves as a robust biomarker for prognostic evaluation in this patient population, providing meaningful implications to guide clinical decision-making in the context of OC.

**Systematic Review Registration:**

https://www.crd.york.ac.uk/PROSPERO/view/CRD420251116811, identifier CRD420251116811.

## 1 Introduction

Ovarian cancer (OC) represents a heterogeneous malignancy, accounting for approximately 310,000 new diagnoses and up to 200,000 deaths globally each year (1). Due to the anatomical position of the ovaries, combined with early symptoms that are often mild or nonspecific, and the lack of effective early detection methods, about 70% of ovarian cancer cases are identified at an advanced stage (2). Primary surgical resection combined with adjuvant chemotherapy continues to be the standard curative therapy for individuals with ovarian cancer. However, treatment efficacy and prognosis remain unsatisfactory. Almost 50% of patients experience relapse within 16 months after treatment, and approximately 70% eventually experience recurrence (3). Patients diagnosed with ovarian cancer exhibit five-year cancer-specific survival rates of 89%, 71%, 41%, and 20% across stages I to IV, respectively (4, 5).

Growing evidence highlights the complex relationship between nutrition and cancer prognosis, prompting continued research into this interaction across multiple malignancies, including ovarian cancer. Besides clinicopathologic and treatment-related factors, immunonutritional status plays a significant role in determining prognosis in ovarian cancer patients (6). Around 70% of patients with ovarian cancer, notably those in advanced stages, suffer from malnutrition caused by factors including a high catabolic state, malignant bowel obstruction, and reduced appetite (7–9). Nutritional deprivation negatively affects treatment efficacy and clinical outcomes in ovarian cancer patients. Malnutrition weakens the immune response, raises the risk of postoperative infections, reduces chemotherapy tolerance, and worsens survival rates (10–12). The prognostic nutritional index (PNI) was initially developed to evaluate the risk of postoperative complications (13). It is clinically applied to evaluate and reflect patients’ nutritional status and inflammatory response, particularly the body’s nutritional condition. The PNI is calculated using the formula: 10 × albumin (g/dl) + 0.005 × total lymphocyte count (per mm³). Recent publications have increasingly demonstrated that PNI is closely linked to clinical outcomes, such as survival and treatment response, across various cancer types. Enrolling 224 patients with early-stage epithelial ovarian cancer and 698 with late-stage disease, Liu et al. demonstrated that high PNI served as an independent protective factor for both PFS and OS in the early-stage group, while among patients with advanced disease, PNI was an independent prognostic factor for OS but had no significant effect on PFS (14). Similarly, Zhang et al. studied 237 ovarian cancer patients who underwent cytoreductive surgery followed by platinum-based chemotherapy and found that a lower PNI was significantly linked to platinum resistance, as well as poorer overall survival (OS) and progression-free survival (PFS), especially in stage III patients (15).

In 2022, Tan et al. performed a comprehensive meta-analysis encompassing 12 studies from 2016 to 2022 to assess the association between PNI and prognosis in patients with ovarian cancer (16). A large number of new studies had emerged since the study by Tan et al. (14, 17–20), and the conclusions of these new studies still existed a controversy. The optimal cut-off value is inconsistent in different studies, and the prognostic relevance of PNI in different pathological subtypes or disease stages is contradictory. The significant heterogeneity in study design and population might be responsible for the inconsistent results of prior studies. Therefore, this systematic review and meta-analysis was conducted with the objective of including the latest published clinical studies based on the previous meta-analysis and updating the data, so as to obtain the latest and most comprehensive evidence to verify the prognostic value of PNI in patients with ovarian cancer. In addition, we included DSS, an indicator that has not been systematically analyzed or underrepresented in previous meta-analyses. Our study had stricter inclusion criteria to reduce heterogeneity due to study-design flaws. And subgroup analysis was conducted for the key clinical factors that were not fully explored in the early stage, such as age and PNI cutoff value.

## 2 Materials and methods

### 2.1 Literature search

This research was conducted in line with the PRISMA 2020 reporting standards (21), with the protocol officially recorded in the International Prospective Register of Systematic Reviews (PROSPERO: CRD420251116811). The search strategy, including both subject headings and keywords, was independently devised by two investigators, ZT and HBT. The literature search was conducted across multiple databases—PubMed, Embase, the Cochrane Library, and Web of Science —covering records from database inception through July 8, 2025. The search employed a broad range of keywords including:”Ovarian Neoplasms,” “Ovarian Neoplasm,” “Ovary Neoplasms,” “Ovary Neoplasm,” “Ovarian Cancer,” “Ovary Cancers,” “Cancer of Ovary,” “Cancer of the Ovary,” “Ovarian Cance,” “Ovarian Cancers,” “prognostic nutritional index,” and “PNI.” **Table 1** provides a comprehensive overview of the literature search strategy.

**Table 1:** Detailed search strategy in four databases.

Database	Search strategy
Pubmed-224	(("Ovarian Neoplasms"[Mesh]) OR (((((((((Ovarian Neoplasm) OR (Ovary Neoplasms)) OR (Ovary Neoplasm)) OR (Ovary Cancer)) OR (Ovary Cancers)) OR (Cancer of Ovary)) OR (Cancer of the Ovary)) OR (Ovarian Cancer)) OR (Ovarian Cancers))) AND ((prognostic nutritional index) OR (PNI))
Embase-73	((Ovarian Neoplasms or (Ovarian Neoplasm or Ovary Neoplasms or Ovary Neoplasm or Ovary Cancer or Ovary Cancers or Cancer of Ovary or Cancer of the Ovary or Ovarian Cancer or Ovarian Cancers)) and (prognostic nutritional index or PNI)).af.
Web of Science-84	((Ovarian Neoplasms) OR (((((((((Ovarian Neoplasm) OR (Ovary Neoplasms)) OR (Ovary Neoplasm)) OR (Ovary Cancer)) OR (Ovary Cancers)) OR (Cancer of Ovary)) OR (Cancer of the Ovary)) OR (Ovarian Cancer)) OR (Ovarian Cancers))) AND ((prognostic nutritional index) OR (PNI)) (Topic)
Chochrane-0	((Ovarian Neoplasms or (Ovarian Neoplasm or Ovary Neoplasms or Ovary Neoplasm or Ovary Cancer or Ovary Cancers or Cancer of Ovary or Cancer of the Ovary or Ovarian Cancer or Ovarian Cancers)) and (prognostic nutritional index or PNI)).af.

### 2.2 Study selection

Eligibility for inclusion was based on meeting the following criteria: (1) patients had a confirmed diagnosis of ovarian cancer (OC) confirmed by pathological examination; (2) studies evaluated the prognostic effect of PNI on overall survival (OS), progression-free survival (PFS), cancer-specific survival (CSS), or disease-specific survival (DSS); (3) studies reported hazard ratios (HR) with 95% confidence intervals (CI), either directly or calculable from accessible data; (4) patients were divided into high-PNI and low-PNI groups based on defined cut-off values; (5) studies were fully published.

Conversely, studies were excluded if they fulfilled any of the following criteria: (1) comments, reviews, meeting abstracts, letters, or case reports; (2) lack of sufficient data to calculate HR and 95% CI; (3) lack of survival outcome data; (4) overlapping or duplicated data.

ZT and HBT independently screened titles and abstracts, examined the full articles, and determined study eligibility. Discrepancies were resolved through mutual agreement.

### 2.3 Data extraction

Data extraction was performed independently by two researchers, ZT and HBT. Any disagreements were resolved through consensus among all co-authors. Collected data included first author’s name, publication year, study location, study duration, study design, sample size, patient age, treatment method, timing of detection, cut-off value, follow-up duration, and HRs (95% CIs) for OS, PFS, CSS, and DSS.

### 2.4 Quality assessment

The Newcastle-Ottawa Quality Assessment Scale (NOS) was applied to evaluate the quality of studies included in our meta-analysis. Studies were assessed on three domains: selection, comparability, and outcomes, with a maximum score of nine points (22). Publications with scores ranging from 7 and 9 were considered high quality (23).

### 2.5 Statistical analysis

Combined HRs and 95% CIs were calculated to determine the prognostic relevance of PNI in OC patients. The presence of heterogeneity was examined using Cochran’s Q test alongside Higgins I² statistic (24). Significant heterogeneity was indicated by I²>50% or P<0.1. Data from all studies were combined using a random-effects model. With each study sequentially excluded, sensitivity analysis was used to explore the influence of single studies on the overall effect size. Additionally, we performed a subgroup analysis of OS based on age, PNI cut-off, and geographic region to assess outcome consistency and potential heterogeneity sources. Funnel plots and Egger’s tests were used to examine publication bias. Statistical significance was set at p < 0.05. Statistical analyses were performed with STATA 15.0 and Review Manager 5.4.

## 3 Results

### 3.1 Study characteristics

**Figure 1:**
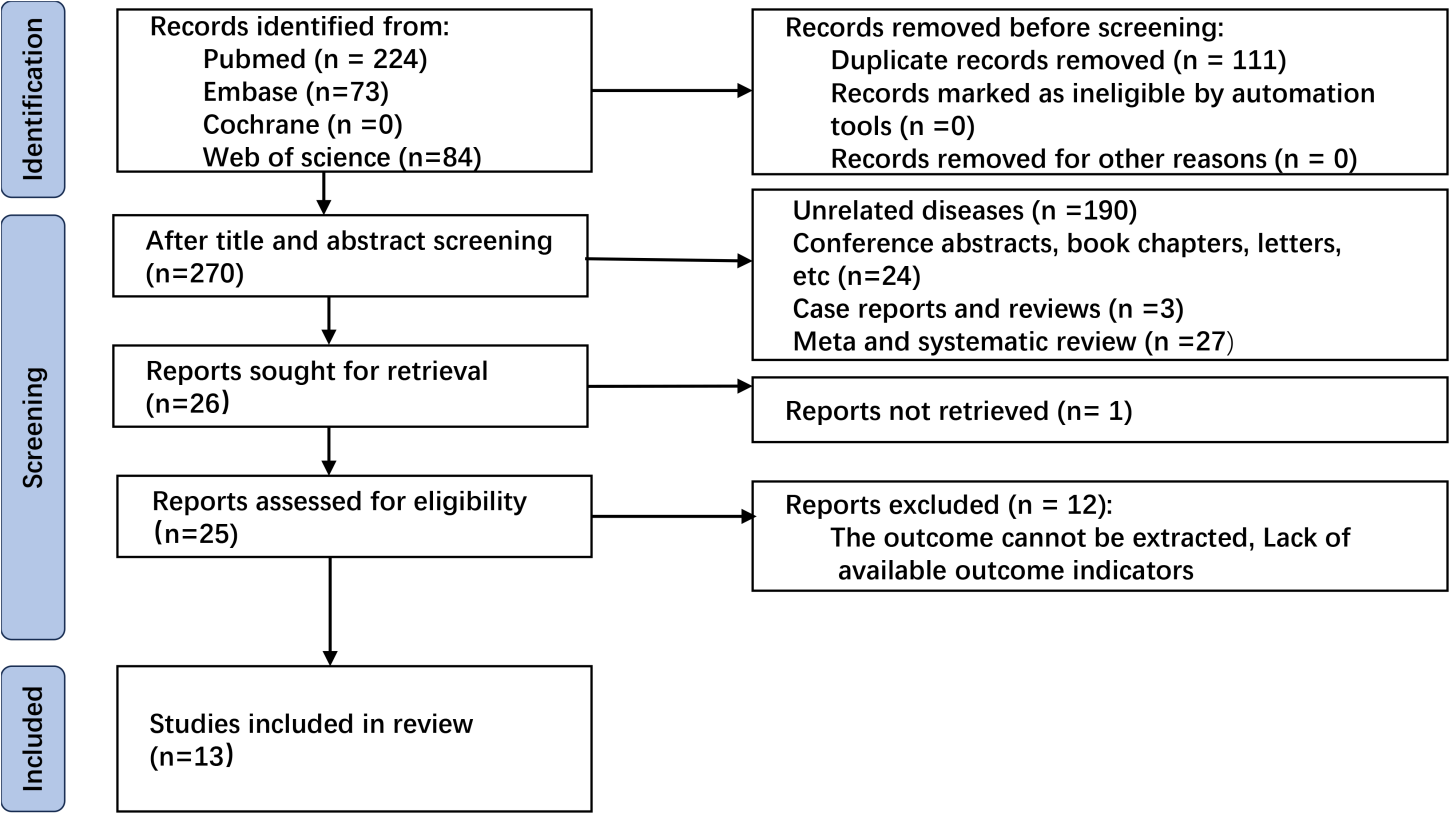
Flowchart of literature screening.

The initial database search retrieved 381 articles, of which 111 duplicates were removed. After screening titles and abstracts, we excluded 244 studies from the analysis. Full texts of 26 articles were reviewed, and 13 were excluded mainly due to lack of sufficient survival data. Finally, 13 studies comprising 5129 patients (14, 15, 17–20, 25–31) were included in our analysis (**Figure 1**).

Within the thirteen included studies, two were conducted in Japan, two in Turkey, and one in Thailand, while the remaining eight originated from China. Notably, two studies (17, 26) included two comparative groups each, and one study (14) included three comparative groups, resulting in a total of seventeen comparative groups. All comparative groups were retrospective (14, 15, 17–20, 25–31). All comparative groups were published in English between 2016 and 2025. Each study categorized patients into high-PNI and low-PNI groups. Regarding PNI assessment, fourteen studies examined its prognostic impact on OS, eight investigated its association with PFS, two assessed its relevance for DSS, and two explored its prognostic value for CSS. A summary of the key characteristics of the included studies is provided in **Table 2**.

**Table 2:** Summary of the key characteristics of the included studies.

Author, Year	Study period	Region	Study design	Population	Treatment method	No. of patients	Mean/median age	Mean/median BMI	FIGO stage	PNI cut-off (measuring method)
Zhang, 2025a	2022-2023	China	Retrospective cohort	ovarian cancer	surgery (PDS or IDS) and chemotherapy	319	/	/	I-IV	46.1 (X-tile)
Zhang, 2025b	2022-2023	China	Retrospective cohort	ovarian cancer	surgery (PDS or IDS) and chemotherapy	319	/	/	I-IV	51.2 (X-tile)
Liu, 2017	2006-2014	China	Retrospective cohort	ovarian cancer	hysterectomy, bilateral salpingo-oophorectomy, pelvic and/or paraaortic lymphadenectomy,appendectomy, and omentectomy. Patients with stage Ic to IV disease received platinum-based chemotherapy following surgery	200	53	/	I-IV	48 (ROC analysis)
Komura, 2019a	2007-2016	Japan	Retrospective cohort	epithelial ovarian cancer	primary cytoreductive surgery or/and neo-adjuvant chemotherapy	164	51	/	I-II	44.7 (ROC analysis)
Komura, 2019b	2007-2016	Japan	Retrospective cohort	epithelial ovarian cancer	primary cytoreductive surgery or/and neo-adjuvant chemotherapy	144	51	/	III-IV	42.9 (ROC analysis)
Miao, 2016	2005-2010	China	Retrospective cohort	epithelial ovarian cancer	comprehensive surgery and djuvant chemotherapy	344	55(45-84)	/	I-IV	45 (ROC analysis)
Han, 2024	2014-2021	China	Retrospective cohort	serous ovarian cancer	surgery and chemotherapy	133	56.68 ± 10.016	/	I-IV	45.5 (ROC analysis)
Zheng, 2018	2005-2013	China	Retrospective cohort	high-grade serous ovarian cancer	primary staging or debulking surgery and platinum-based chemotherapy	875	56(30–90)	22.8(15.6–37.3)	I-IV	45.45 (citation of prior work)
Zhang, 2018	2007-2015	China	Retrospective cohort	ovarian cancer	cytoreductive surgery and platinum-based chemotherapy	237	50(24-76)	/	I-IV	47.2 (Cutoff Finder)
Yoshikawa, 2020	2005-2017	Japan	Retrospective cohort	stage I–II ovarian clear cell carcinoma	primary surgery and adjuvant chemotherapy	82	53.45 ± 10.18	21.23 ± 3.63	I-II	46.5 (ROC analysis)
Chen, 2025	2016-2020	China	Retrospective cohort	epithelial ovarian cancer	fully staged surgery	154	55(49–64)	/	I-IV	48.98 (ROC analysis)
Liu, 2025a	2012-2023	China	Retrospective cohort	early-stage epithelial ovarian cancer	comprehensive staged surgery or debulking surgery and postoperative chemotherapy	224	52 (47, 59)	22.0 (20.3, 24.1)	I-IIA	47.47 (R package “survminer”)
Liu, 2025b	2012-2023	China	Retrospective cohort	late-stage epithelial ovarian cancer	comprehensive staged surgery or debulking surgery and postoperative chemotherapy	698	52 (47, 59)	22.0 (20.3, 24.1)	IIB-IV	46 (R package “survminer”)
Liu, 2025c	2012-2023	China	Retrospective cohort	late-stage epithelial ovarian cancer	comprehensive staged surgery or debulking surgery and postoperative chemotherapy	698	52 (47, 59)	22.0 (20.3, 24.1)	IIB-IV	47.76 (R package “survminer”)
Garbioglu, 2023	2012-2019	Turkey	Retrospective cohort	high-grade serous ovarian cancer	paclitaxel and carboplatin chemotherapy	80	57.21 ± 10.49	/	III-IV	47.5 (median split)
Prueksaritanond, 2024	2008-2019	Thailand	Retrospective cohort	epithelial ovarian clear cell cancer	primary cytoreductive surgery and adjuvant chemotherapy	290	53.3 ± 9.4	23.6 ± 4.6	I-IV	50 (the mean value)
Karakaş, 2022	2015-2020	Turkey	Retrospective cohort	ovarian cancer	surgery and chemotherapy	168	55.7 ± 10.5	32.4 ± 7.6	I-IV	45.98 (ROC analysis)

### 3.2 Study quality

All thirteen studies received NOS scores ranging from 7 to 8, reflecting high quality (**Table 3**).

**Table 3:** Quality evaluation of the eligible studies with Newcastle–Ottawa scale.

Study	Selection				Comparability		Outcome		
	Representative-ness	Selection of non-exposed	Ascertainment of exposure	Outcome not present at start	Comparability on most important factors	Comparability on other risk factors	Assessment of outcome	Long enough follow-up (median≥1 year)	Adequacy (completeness) of follow-up
Zhang,2025	*	*	*	*	–	–	*	*	*
Liu,2017	*	*	*	*	–	–	*	*	*
Komura,2019	*	*	*	*	–	–	*	*	*
Miao,2016	*	*	*	*	–	–	*	*	*
Han,2024	*	*	*	*	–	–	*	*	*
Zheng,2018	*	*	*	*	–	–	*	*	*
Zhang,2018	*	*	*	*	–	–	*	*	*
Yoshikawa,2020	*	*	*	*	*	–	*	*	*
Chen,2025	*	*	*	*	–	–	*	*	*
Liu,2025	*	*	*	*	*	–	*	*	*
Garbioglu,2023	*	*	*	*	*	–	*	*	*
Prueksaritanond,2024	*	*	*	*	–	–	*	*	*
Karakaş,2022	*	*	*	*	–	–	*	*	*

*indicates criterion met; - indicates significant of criterion not met.

### 3.3 Meta-analysis results

#### 3.3.1 PNI and OS

**Figure 2:**
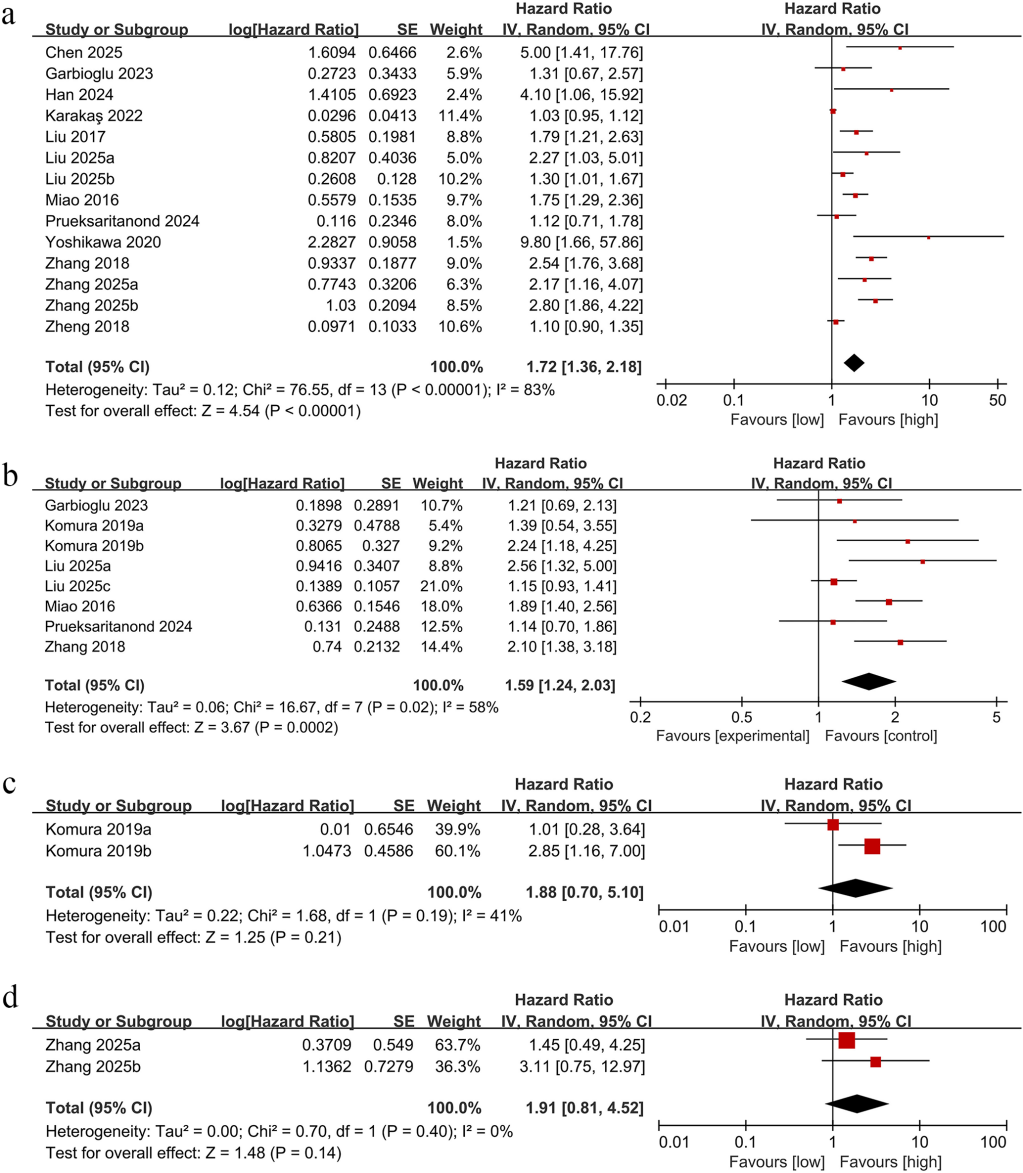
**(A)** Forest plots for the association between PNI and OS; **(B)** Forest plots for the association between PNI and PFS; **(C)** Forest plots for the association between PNI and DSS; **(D)** Forest plots for the association between PNI and CSS.

The association between PNI and OS was analyzed across fourteen comparative groups including 4,123 participants. Due to significant heterogeneity among studies (I² = 83%, p < 0.00001), a random-effects model was applied (**Figure 2A**). The analysis indicated that decreased PNI was linked to reduced OS in OC patients (HR = 1.72, 95% CI:1.36-2.18; p<0.00001, **Figure 2A**).

Subgroup analysis of OS was conducted based on age, PNI cut-off value, and region. The analysis demonstrated that the prognostic effect of PNI for OS was not observed in the subgroup of patients older than 55 years old (p=0.31; **Table 4**), in Turkey (p=0.42; **Table 4**), and in Thailand (p=0.62; **Table 4**), but it remained significant in the other subgroups. Moreover, there was low heterogeneity in the subgroup of patients older than 55 years (I²=36%), and the Turkey subgroup (I²=0%) indicated no significant heterogeneity.

**Table 4:** Pooled HRs for OS and PFS in subgroup analyses.

Subgroup	OS				PFS			
	Comparative groups	HR [95%CI]	*P* value	*I* ^2^	Comparative groups	HR [95%CI]	*P* value	*I* ^2^
Total	14	1.72 [1.36, 2.18]	0.00001	83%	8	1.59 [1.24, 2.03]	0.0002	58%
Age								
≤55year	8	1.83 [1.39, 2.41]	0.00001	64%	7	1.65 [1.25, 2.16]	0.004	63%
>55year	4	1.09 [0.93, 1.28]	0.31	36%	1	1.21 [0.69, 2.13]	0.51	/
PNI cut-off								
≤46	5	1.27 [1.02, 1.57]	0.03	76%	3	1.90 [1.46, 2.47]	0.00001	0%
>46	9	2.09 [1.57, 2.78]	0.00001	54%	5	1.47 [1.07, 2.01]	0.02	62%
Region								
China	10	1.92 [1.47, 2.50]	0.00001	75%	4	1.75 [1.20, 2.54]	0.004	78%
Japan	1	9.80 [1.66, 57.86]	0.01	/	2	1.92 [1.13, 3.27]	0.02	0%
Turkey	2	1.03 [0.95, 1.12]	0.42	0%	1	1.21 [0.69, 2.13]	0.51	/
Thailand	1	1.12 [0.71, 1.78]	0.62	/	1	1.14 [0.70, 1.86]	0.53	/

#### 3.3.2 PNI and PFS

Eight studies provided data on PNI and PFS, revealing that lower PNI correlated with reduced PFS in OC patients (HR = 1.59,95% Cl:1.24–2.03; p=0.0002, **Figure 2B**), with substantial heterogeneity detected (I²=58%, p=0.02, **Figure 2B**).

Subgroup analysis of PFS was performed based on age, PNI cut-off value, and region. Our subgroup analyses revealed that no significant prognostic effect was observed with patients over the age of 55 (p=0.51; **Table 4**), Turkey (p=0.51; **Table 4**), and Thailand (p=0.53;**Table 4**), but there was a significant association between the remaining subgroups and PFS. There was no significant heterogeneity for Japan (I²=0) and for the PNI ≤46 (I²=0).

#### 3.3.3 PNI and DSS

Only two studies explored the relationship between PNI and DSS. With low heterogeneity (I²=41%;P = 0.19, **Figure 2C**), no significant prognostic effect was observed with DSS (HR = 1.88,95% Cl:0.70–5.10; p=0.21, **Figure 2C**).

#### 3.3.4 PNI and CSS

Just two groups of studies investigated the effect of PNI on CSS. No notable heterogeneity was observed (I²= 0%, p=0.40, **Figure 2D**), and PNI was not significantly associated with CSS (HR = 1.91,95% Cl:0.81–4.52; p=0.14, **Figure 2D**).

#### 3.3.5 Sensitivity analysis

**Figure 3:**
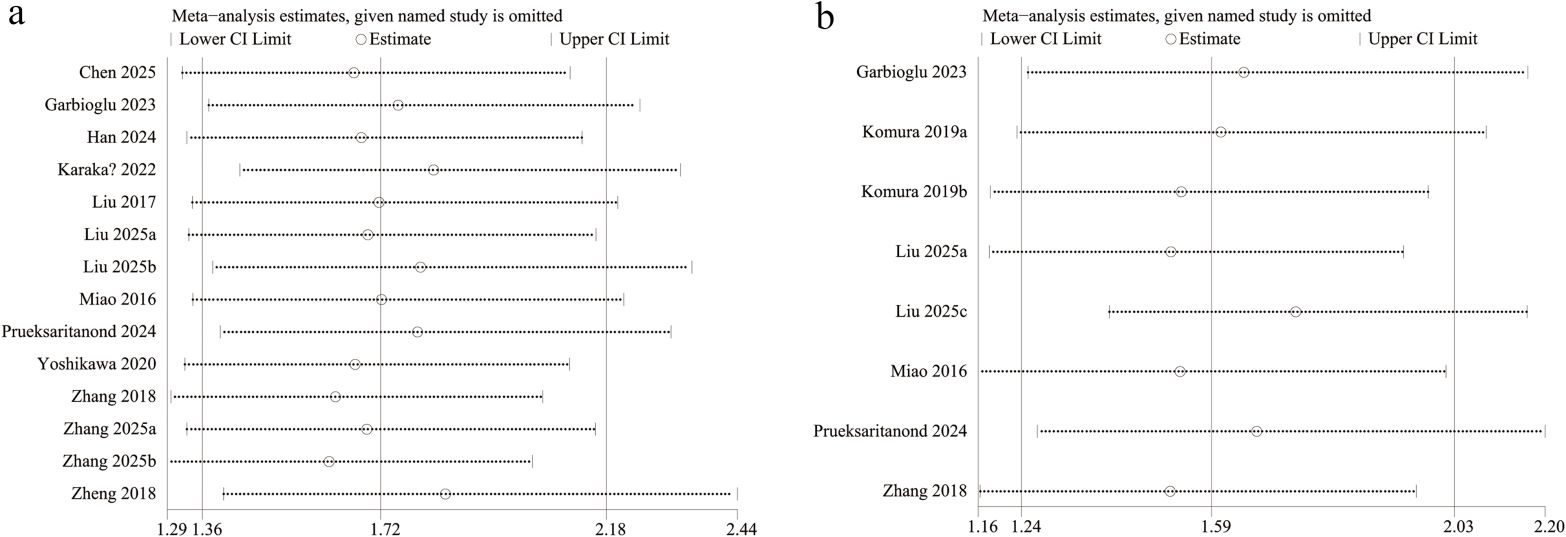
Sensitivity analysis of **(A)** OS and **(B)** PFS.

We conducted a sensitivity analysis to evaluate the reliability of our findings regarding PNI’s role in patient outcomes. Sequential removal of each study showed consistent effect sizes, indicating that no individual study had an excessive impact on OS (**Figure 3A**) and PFS (**Figure 3B**), supporting the robustness of the analysis.

### 3.5 Publication bias

**Figure 4:**
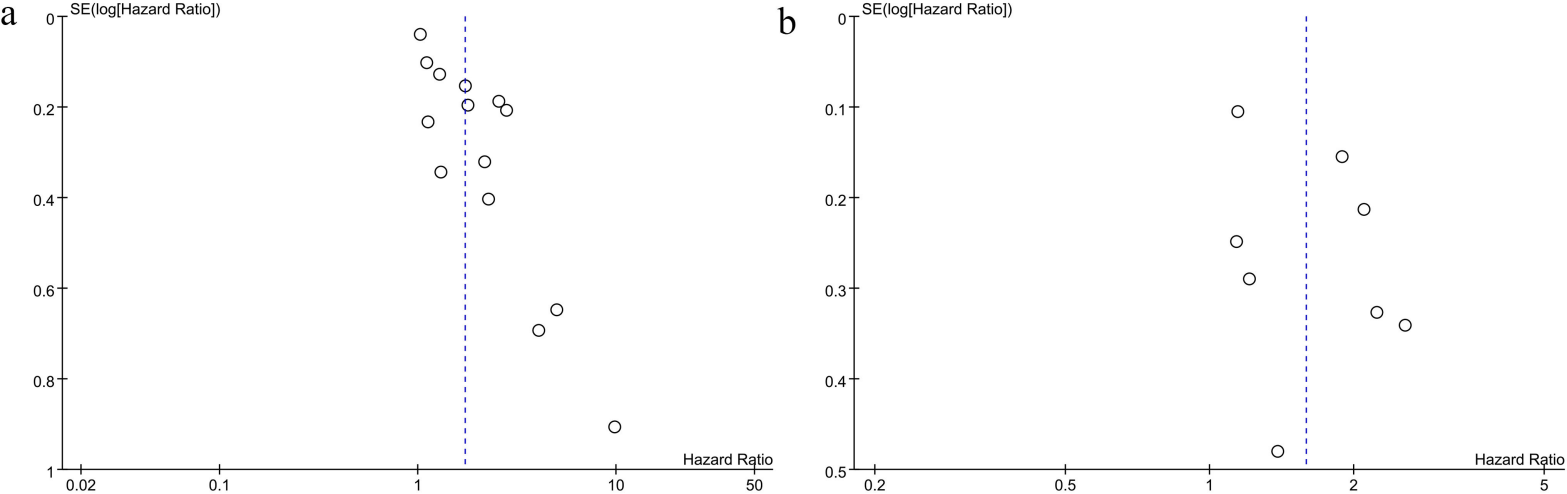
Funnel plot for the evaluation of publication bias for **(A)** OS and **(B)** PFS.

Publication bias was evaluated through funnel plots and Egger’s test. The funnel plot indicated notable publication bias in the OS meta-analysis (**Figure 4A**). However, no evidence of publication bias was detected for PFS (**Figure 4B**). Egger’s test showed no significant publication bias for PFS (p = 0.25), but pointed to potential bias in the OS analysis (Egger: p = 0.0001).

## 4 Discussion

There exists a strong association between nutritional status and tumor development. According to Laky et al., approximately 20% of newly diagnosed gynecologic cancer patients exhibit signs of malnutrition (32). Furthermore, more than 20% of cancer-related deaths are attributed to malnutrition rather than the direct effects of the malignancy itself (33). Due to the special biological characteristics of ovarian cancer, the abnormal metabolism of tumor tissue and abdominal complications continuously consume a large amount of energy and protein. Patients with ovarian cancer are prone to malnutrition and cachexia due to the metabolic impact of tumor burden, malignant ascites, and small bowel obstruction (34). Given the critical role of nutritional status in ovarian cancer progression and prognosis, identifying indicators for accurate assessment of patients’ nutritional and immune status is paramount.

Our meta-analysis indicated the prognostic significance of PNI for both PFS and OS in ovarian cancer patients. Decreased PNI correlated with shorter OS and PFS, indicating a poorer prognosis. These results emphasize the possible role of PNI as a valuable prognostic indicator. PNI can be combined with traditional prognostic factors, such as FIGO stage, pathological differentiation, and CA125 levels, to improve the accuracy of prognosis assessment in ovarian cancer patients. This combined approach can help clinicians identify high-risk individuals who may benefit from more intensive adjuvant therapy or closer postoperative follow-up. However, likely due to the limited sample size, no significant predictive value of PNI was observed for DSS or CSS. Additional research is required to clarify the prognostic value of PNI for DSS and CSS. The sensitivity analysis confirmed the robustness and reliability of our findings, indicating high-quality evidence. While no publication bias was observed for PFS, significant bias was detected for OS. In studies assessing the association between PNI and OS in ovarian cancer, such publication bias may substantially distort conclusions. Positive results are more likely to be published, favoring studies that report significant OS benefits associated with high PNI, whereas neutral or negative findings often remain unpublished. This selective publication can systematically overestimate the prognostic value of PNI. Furthermore, OS in ovarian cancer is influenced by multiple factors, including tumor stage, pathological subtype, and treatment response, which may lead to an inflated perception of PNI’s independent prognostic significance. Furthermore, publication bias significantly undermines the internal validity of this study. The overrepresentation of positive results may lead to an overestimation of the combined Hazard Ratio, potentially exaggerating the conclusion that PNI is a strong prognostic indicator. Such bias can diminish the overall level and quality of evidence by compromising its authenticity, reliability, and generalizability. Therefore, further validation in additional studies is warranted. Beyond the impact of publication bias, our findings are consistent with the 2022 meta-analysis by Tan et al. (16), and we have confirmed the predictive value of PNI for OS and PFS in ovarian cancer patients using a larger sample size.

In the subgroup analysis, PNI lost its predictive value for both OS and PFS in subgroups of patients aged over 55 years, as well as in the Turkey and Thailand subgroups. Conversely, PNI retained significant predictive value in the remaining subgroups. These findings suggest that PNI-based prognostication may be more suitable for individuals aged 55 years or younger. This phenomenon may be related to the progressive deterioration of nutritional status with advancing age. Aging can impair nutrient absorption due to a decline in myenteric plexus neurons that regulate digestion and a reduction in small intestinal surface area resulting from villus degeneration (35). This physiological decline not only reduces the bioavailability of essential nutrients but also increases the risk of malnutrition, which is further aggravated by age-related changes in digestive enzyme secretion and alterations in gut microbiota composition. Furthermore, etiological analyses indicate that immunosenescence and protein-energy malnutrition (PEM) are major contributing factors, often accompanied by widespread deficiencies in micronutrients such as vitamin D, zinc, and vitamin E in individuals over 75 years of age (36). The coexistence of immunosenescence and PEM creates a self-perpetuating vicious cycle: impaired immunity increases susceptibility to infections and chronic low-grade inflammation (inflammaging), which in turn exacerbates nutritional losses and malabsorption, further weakening immune function. Additionally, more than half of elderly cancer patients take at least five medications, and potential drug–drug interactions may contribute to reduced physical function (37, 38). Over time, these drug-related disruptions to intake, absorption, and metabolism contribute to a progressive decline in nutritional status, which in turn further compromises physical function and resilience to cancer treatments. In addition, chronic kidney disease, diabetes, and other complications in elderly patients with cancer further affect nutritional status. Therefore, given that PNI serves as a nutritional indicator, its application in predicting OS and PFS in elderly patients with compromised nutritional status may yield less reliable results due to the interference of non-tumor-related factors on platelet and lymphocyte levels. In clinical practice, it is recommended to integrate nutritional assessment, comprehensive geriatric assessment, and dynamic monitoring to achieve a more accurate evaluation of prognosis for this specific patient population. Future studies should conduct subgroup analyses specifically targeting malnourished elderly patients to determine the optimal application and potential modifications of the PNI. The limited number of studies in the Turkish and Thai subgroups may have contributed to the observed loss of predictive value. Additional studies are needed to confirm these results and establish the predictive utility of PNI across populations of diverse regions.

PNI is determined based on serum albumin concentration and the total number of lymphocytes. Albumin is an important form of nutrient reserve in the body, and its level directly reflects protein intake, synthesis, and consumption. Albumin, the most abundant protein in plasma, has been reported to have a negative correlation with pro-inflammatory and angiogenic cytokine levels in cancer patients (39). Low serum albumin levels are commonly observed in malnourished individuals. In malnutrition, the decrease in albumin level not only indicates insufficient reserve, but also may affect the body’s metabolism and organ function, and further aggravate nutritional deterioration. Moreover, low albumin levels may indicate inflammation driven by cytokines, including interleukin-6 (IL-6) and tumor necrosis factor (40). Lymphocytes are the core cells of the immune system. Malnutrition can suppress immune function through a variety of mechanisms, such as reduced cytokine synthesis and decreased efficiency of antibody production, and a reduced lymphocyte number is a direct manifestation of reduced immune function. Therefore, TLC not only reflects nutrient intake, but also indicates the effect of nutrition on the body’s defense function. The inflammatory process, along with the infiltration of endothelial immune cells in ovarian cancer, can result in a decreased lymphocyte count (41). In addition, evidence indicates that inflammation may reduce the absolute lymphocyte count (ALC). In murine models, IL-6 has been shown to inhibit lymphopoiesis, resulting in increased production of myeloid cells (42). Notably, reduced ALC is strongly associated with higher serum concentrations of IL-6 and IL-2 in patients with soft tissue sarcoma (43).

There is a close relationship between nutritional status and tumors. Inflammatory factors released by tumors aggravate nutritional consumption, which can occur as immunosuppression and hypoalbuminemia. Many studies indicate that malnourished cancer patients often experience compromised immune systems, making them more susceptible to infections and treatment-related complications, such as delayed wound healing. Worse nutritional status in patients is associated with reduced immune function, which can consequently accelerate disease progression (44). Additionally, malnutrition may reduce the effectiveness of radiotherapy and chemotherapy, ultimately leading to a poorer prognosis. In contrast, good nutritional status serves as the foundation for maintaining immune function and withstanding antitumor therapies, such as surgery, radiotherapy, and chemotherapy. Nutritional support therapy can significantly enhance the prognosis of cancer patients (45). It can enhance the capacity of immune cells to target and destroy tumor cells, improve tolerance to treatment, and potentially prolong patient survival. PNI accurately quantifies the interaction between nutritional and immune status using albumin and ALC. Its design logic accounts for the multi-dimensional impacts of nutrition on the body, and it has been clinically validated for its effectiveness in identifying malnutrition and predicting nutrition-related adverse outcomes. Therefore, PNI is a practical tool for the clinical assessment of patients’ nutritional status, particularly for those at high nutritional risk, which has important clinical significance for the prognosis evaluation of ovarian cancer patients.

While our meta-analysis yields valuable insights, it is necessary to acknowledge several limitations that merit careful consideration. First, the overwhelming majority of publications included in our analysis were conducted in Asian regions, primarily China and Japan. As such, our conclusions should be contextualized within this geographic framework, and caution is advised when extending these results to patient populations in Europe, the Americas, Africa, or other regions. Further investigations are needed to verify the prognostic significance of PNI in non-Asian ovarian cancer patients. In the future, it will be essential to conduct additional international, multicenter studies incorporating more diverse populations across various regions. This expanded geographical representation of samples would enable further validation and refinement of our findings. Second, the majority of included publications employed a retrospective design instead of a prospective one. This retrospective approach may introduce confounding factors that could affect the robustness of our findings. Retrospective studies are inherently prone to selection bias, information bias, and heterogeneity. These limitations may hinder the establishment of causality and restrict the generalizability of the findings. Additionally, a further limitation lies in the inconsistency of PNI cut-off values among the included publications, which varied from 42.9 to 51.2. Different studies employed diverse methods to determine the PNI cut-off value, which inherently brings an impact to the results. Such inconsistencies in threshold values may contribute to heterogeneity in the meta-analysis, underscoring the need of defining a standardized PNI cut-off in future studies to enhance reliability and comparability. And due to insufficient and inconsistent reporting of data across the included studies, a comprehensive exploration of heterogeneity sources related to treatment regimens and tumor stages remains limited. Moreover, due to the limited sample size of DSS and CSS, the analysis results are mainly exploratory analysis, which cannot obtain accurate and reliable results and exclude false negative results, and further studies are needed to explain it. Finally, significant publication bias was detected for OS outcomes, highlighting the necessity for further validation in subsequent related research.

## 5 Results

This study demonstrates the potential predictive efficacy of PNI for OS and PFS in ovarian cancer patients, with a lower PNI correlating with reduced OS and PFS durations. Subgroup analyses revealed that age and geographical region may influence PNI’s predictive validity. Since most of the analyzed studies used a retrospective design and were mainly performed in Asian populations, concerns regarding heterogeneity and publication bias persist. Future research should prioritize international multicenter prospective studies to validate PNI’s predictive utility in ovarian cancer, thereby facilitating the development of a more precise nutrition-based prognostic model for ovarian cancer patients.

## Data Availability

The original contributions presented in the study are included in the article/supplementary material. Further inquiries can be directed to the corresponding author.

## References

[1] Hollis RL . Molecular characteristics and clinical behavior of epithelial ovarian cancers. Cancer Lett. (2023) 555:216057. doi: 10.1016/j.canlet.2023.216057, PMID: 36627048

[2] Siegel RL Giaquinto AN Jemal A . Cancer statistics, 2024. CA Cancer J Clin. (2024) 74:12–49. doi: 10.3322/caac.21820, PMID: 38230766

[3] Pignata S CC S Du Bois A Harter P Heitz F . Treatment of recurrent ovarian cancer. Ann Oncol. (2017) 28:viii51–viii6. doi: 10.1093/annonc/mdx441, PMID: 29232464

[4] Hurwitz LM Pinsky PF Trabert B . General population screening for ovarian cancer. Lancet. (2021) 397:2128–30. doi: 10.1016/S0140-6736(21)01061-8, PMID: 33991478

[5] Torre LA Trabert B DeSantis CE Miller KD Samimi G Runowicz CD . Ovarian cancer statistics, 2018. CA Cancer J Clin. (2018) 68:284–96. doi: 10.3322/caac.21456, PMID: 29809280 PMC6621554

[6] Chien J Poole EM . Ovarian cancer prevention, screening, and early detection: report from the 11th biennial ovarian cancer research symposium. Int J Gynecol Cancer. (2017) 27:S20–s2. doi: 10.1097/IGC.0000000000001118, PMID: 29278600 PMC6154781

[7] Hébuterne X Lemarié E Michallet M de Montreuil CB Schneider SM Goldwasser F . Prevalence of malnutrition and current use of nutrition support in patients with cancer. JPEN J Parenter Enteral Nutr. (2014) 38:196–204. doi: 10.1177/0148607113502674, PMID: 24748626

[8] Hertlein L Kirschenhofer A Fürst S Beer D Göß C Lenhard M . Malnutrition and clinical outcome in gynecologic patients. Eur J Obstet Gynecol Reprod Biol. (2014) 174:137–40. doi: 10.1016/j.ejogrb.2013.12.028, PMID: 24485666

[9] Laky B Janda M Cleghorn G Obermair A . Comparison of different nutritional assessments and body-composition measurements in detecting malnutrition among gynecologic cancer patients. Am J Clin Nutr. (2008) 87:1678–85. doi: 10.1093/ajcn/87.6.1678, PMID: 18541556

[10] Goins EC Weber JM Truong T Moss HA Previs RA Davidson BA . Malnutrition as a risk factor for post-operative morbidity in gynecologic cancer: Analysis using a national surgical outcomes database. Gynecol Oncol. (2022) 165:309–16. doi: 10.1016/j.ygyno.2022.01.030, PMID: 35241292

[11] Nomoto N Tate S Arai M Iizaka S Mori C Sakurai K . Pretreatment nutritional status in combination with inflammation affects chemotherapy interruption in women with ovarian, fallopian tube, and peritoneal cancer. Nutrients. (2022) 14:5183. doi: 10.3390/nu14235183, PMID: 36501212 PMC9741349

[12] Rinninella E Fagotti A Cintoni M Raoul P Scaletta G Quagliozzi L . Nutritional interventions to improve clinical outcomes in ovarian cancer: A systematic review of randomized controlled trials. Nutrients. (2019) 11:1404. doi: 10.3390/nu11061404, PMID: 31234395 PMC6627677

[13] Onodera T Goseki N Kosaki G . Prognostic nutritional index in gastrointestinal surgery of malnourished cancer patients. Nihon Geka Gakkai Zasshi. (1984) 85:1001–5. 6438478

[14] Liu X Li M Zhao Y Jiao X Yu Y Li R . The impact of preoperative immunonutritional status on prognosis in ovarian cancer: a multicenter real-world study. J Ovarian Res. (2025) 18:30. doi: 10.1186/s13048-025-01607-4, PMID: 39962572 PMC11831797

[15] Zhang WW Ye B Liang WJ Ren YZ . Preoperative prognostic nutritional index is a powerful predictor of prognosis in patients with stage III ovarian cancer. Sci Rep. (2018) 7:9548. doi: 10.1038/s41598-017-10328-8, PMID: 28842710 PMC5573316

[16] Tan X Chen H . The prognostic value of prognostic nutritional index in patients with ovarian cancer: A systematic review and meta-analysis. Nutr Cancer. (2023) 75:73–81. doi: 10.1080/01635581.2022.2104879, PMID: 35900054

[17] Zhang Y Xing W Liang X Yang Z Ma Y Chen Y . Relationship between nutritional-inflammatory markers and postoperative outcomes in ovarian cancer: a retrospective study. Front Oncol. (2025) 15:1531987. doi: 10.3389/fonc.2025.1531987, PMID: 40134604 PMC11932915

[18] Han Y Lv W Guo J Shang Y Yang F Zhang X . Prognostic significance of inflammatory and nutritional indices for serous ovary cancer. Clin Exp Obstet Gynecol. (2024) 51:5. doi: 10.31083/j.ceog5101005

[19] Chen J Jin L Luo R Zhang X Chen Y Han Z . Predictive value of preoperative systemic immune-inflammation index and prognostic nutrition index in patients with epithelial ovarian cancer. J Ovarian Res. (2025) 18:45. doi: 10.1186/s13048-025-01631-4, PMID: 40055764 PMC11887369

[20] Prueksaritanond N Petchsila K Insin P . The association of preoperative prognostic nutritional index with survival outcome in ovarian clear cell cancer. World J Oncol. (2024) 15:950–9. doi: 10.14740/wjon1963, PMID: 39697428 PMC11650612

[21] Page MJ McKenzie JE Bossuyt PM Boutron I Hoffmann TC Mulrow CD . The PRISMA 2020 statement: an updated guideline for reporting systematic reviews. Syst Rev. (2021) 10:89. doi: 10.1186/s13643-021-01626-4, PMID: 33781348 PMC8008539

[22] Wells G Shea B O’Connell D Peterson J Welch M Losos P . eds. The newcastle-ottawa scale (NOS) for assessing the Quality of Nonrandomized Studies in Meta-Analyses2014. Ottawa: Medicine. (2014).

[23] Mavaddat N Michailidou K Dennis J Lush M Fachal L Lee A . Polygenic risk scores for prediction of breast cancer and breast cancer subtypes. Am J Hum Genet. (2019) 104:21–34. doi: 10.1016/j.ajhg.2018.11.002, PMID: 30554720 PMC6323553

[24] Higgins JP Thompson SG Deeks JJ Altman DG . Measuring inconsistency in meta-analyses. BMJ. (2003) 327:557–60. doi: 10.1136/bmj.327.7414.557, PMID: 12958120 PMC192859

[25] Liu YB Chen SF Zheng CY Ding M Zhang L Wang LG . The prognostic value of the preoperative c-reactive protein/albumin ratio in ovarian cancer. BMC Cancer. (2017) 17:285. doi: 10.1186/s12885-017-3220-x, PMID: 28431566 PMC5399817

[26] Komura N Mabuchi S Yokoi E Shimura K Kawano M Matsumoto Y . Prognostic significance of the pretreatment prognostic nutritional index in patients with epithelial ovarian cancer. Oncotarget. (2019) 10:3605–13. doi: 10.18632/oncotarget.26914, PMID: 31217896 PMC6557203

[27] Miao Y Li S Yan Q Li B Feng Y . Prognostic significance of preoperative prognostic nutritional index in epithelial ovarian cancer patients treated with platinum-based chemotherapy. Oncol Res Treat. (2016) 39:712–9. doi: 10.1159/000452263, PMID: 27855385

[28] Zheng F Hao W Xingzhu J Rui B Xiaojun C Wentao Y . Preoperative prognostic nutritional index is a predictive and prognostic factor of high-grade serous ovarian cancer. Int J Gynecol Cancer. (2018) 28:71. doi: 10.1186/s12885-018-4732-8, PMID: 30200903 PMC6131794

[29] Yoshikawa N Yoshida K Tamauchi S Ikeda Y Nishino K Niimi K . The preoperative prognostic nutritional index for the prediction of outcomes in patients with early-stage ovarian clear cell carcinoma. Sci Rep. (2020) 10:7135. doi: 10.1038/s41598-020-64171-5, PMID: 32346076 PMC7189228

[30] Garbioglu DB Gundogdu E Demir N Dincer M . Evaluation of the relationship of the amount of ascites as measured quantitatively using computed tomography with chemotherapy toxicity in patients with ovarian cancer. Eur J Gynecol Oncol. (2023) 44:46–54. doi: 10.22514/ejgo.2023.078

[31] Karakaş S Demirayak G Önder AB Özdemir İA Comba C Süzen Çaypınar S . The association between the preoperative prognostic nutritional index and the controlling nutritional status score on tumor stage, chemotherapeutic response and overall survival in ovarian cancer. Nutr Cancer. (2022) 74:1770–9. doi: 10.1080/01635581.2021.2022170, PMID: 34989281

[32] Laky B Janda M Bauer J Vavra C Cleghorn G Obermair A . Malnutrition among gynecological cancer patients. Eur J Clin Nutr. (2007) 61:642–6. doi: 10.1038/sj.ejcn.1602540, PMID: 17021596

[33] Ottery FD . Cancer cachexia: prevention, early diagnosis, and management. Cancer Pract. (1994) 2:123–31., PMID: 8055014

[34] García-Luna PP Parejo Campos J Pereira Cunill JL . Causes and impact of hyponutrition and cachexia in the oncologic patient. Nutr Hosp. (2006) 21:10–6., PMID: 16768026

[35] Soenen S Rayner CK Jones KL Horowitz M . The ageing gastrointestinal tract. Curr Opin Clin Nutr Metab Care. (2016) 19:12–8. doi: 10.1097/MCO.0000000000000238, PMID: 26560524

[36] Tannou T Koeberle S Manckoundia P Aubry R . Multifactorial immunodeficiency in frail elderly patients: Contributing factors and management. Med Mal Infect. (2019) 49:167–72. doi: 10.1016/j.medmal.2019.01.012, PMID: 30782449

[37] Turner JP Shakib S Singhal N Hogan-Doran J Prowse R Johns S . Prevalence and factors associated with polypharmacy in older people with cancer. Support Care Cancer. (2014) 22:1727–34. doi: 10.1007/s00520-014-2171-x, PMID: 24584682

[38] Prithviraj GK Koroukian S Margevicius S Berger NA Bagai R Owusu C . Patient characteristics associated with polypharmacy and inappropriate prescribing of medications among older adults with cancer. J Geriatr Oncol. (2012) 3:228–37. doi: 10.1016/j.jgo.2012.02.005, PMID: 22712030 PMC3375830

[39] McMillan DC . The systemic inflammation-based Glasgow Prognostic Score: a decade of experience in patients with cancer. Cancer Treat Rev. (2013) 39:534–40. doi: 10.1016/j.ctrv.2012.08.003, PMID: 22995477

[40] Shioya M Yoshida T Kasai K Furuya R Kato A Mori N . Inflammatory factors for hypoalbuminemia in Japanese peritoneal dialysis patients. Nephrol (Carlton). (2013) 18:539–44. doi: 10.1111/nep.12106, PMID: 23718260

[41] Winkler I Woś J Bojarska-Junak A Semczuk A Rechberger T Baranowski W . An association of iNKT+/CD3+/CD161+ lymphocytes in ovarian cancer tissue with CA125 serum concentration. Immunobiology. (2020) 225:152010. doi: 10.1016/j.imbio.2020.152010, PMID: 33130518

[42] Maeda K Malykhin A Teague-Weber BN Sun XH Farris AD Coggeshall KM . Interleukin-6 aborts lymphopoiesis and elevates production of myeloid cells in systemic lupus erythematosus-prone B6.Sle1.Yaa animals. Blood. (2009) 113:4534–40. doi: 10.1182/blood-2008-12-192559, PMID: 19224760 PMC2680362

[43] Ruka W Rutkowski P Kaminska J Rysinska A Steffen J . Alterations of routine blood tests in adult patients with soft tissue sarcomas: relationships to cytokine serum levels and prognostic significance. Ann Oncol. (2001) 12:1423–32. doi: 10.1023/A:1012527006566, PMID: 11762815

[44] Pintucci G Froum S Pinnell J Mignatti P Rafii S Green D . Trophic effects of platelets on cultured endothelial cells are mediated by platelet-associated fibroblast growth factor-2 (FGF-2) and vascular endothelial growth factor (VEGF). Thromb Hemost. (2002) 88:834–42. doi: 10.1055/s-0037-1613311, PMID: 12428103

[45] Lewis SR Schofield-Robinson OJ Alderson P Smith AF . Enteral versus parenteral nutrition and enteral versus a combination of enteral and parenteral nutrition for adults in the intensive care unit. Cochrane Database Syst Rev. (2018) 6:Cd012276. doi: 10.1002/14651858.CD012276.pub2, PMID: 29883514 PMC6353207

